# A Retrospective Comparison of Radiotherapy and Stenting for the Relief of Dysphagia and Pain in Advanced Esophageal Cancer

**DOI:** 10.7759/cureus.113474

**Published:** 2026-07-27

**Authors:** Asifa Andleeb, Kaneez Fatima, Shahida Nasreen, Mushtaq Ahmad Sofi, Ulfat Ara, Shaheen Nazir, Musaib Mushtaq, Taskeen Mehmood

**Affiliations:** 1 Radiation Oncology, Sher-i-Kashmir Institute of Medical Sciences, Srinagar, IND; 2 Medical Oncology, Sher-i-Kashmir Institute of Medical Sciences, Srinagar, IND; 3 Gastroenterology and Hepatology, Sher-i-Kashmir Institute of Medical Sciences, Srinagar, IND

**Keywords:** dysphagia, esophagus, locally advanced stage, pain, palliation, treatment naïve

## Abstract

Objectives: The objective of this study was to compare the efficacy of external beam radiotherapy (EBRT) and self-expanding metal stents (SEMS) in relieving dysphagia and pain, while also evaluating their treatment-related adverse event profiles in patients with advanced esophageal cancer.

Materials and methods: It is a retrospective review based on observations of 130 patients who presented with locally advanced esophageal cancer and were not fit to receive any curative treatment, and the only intention of treatment was symptom palliation, especially dysphagia and pain.

Results: We observed a significant and comparable improvement in dysphagia scores in both groups. Based on Ogilvie dysphagia scores, the mean pretreatment dysphagia score for the EBRT group was 2.6; for the SEMS group, it was 3; and respective dysphagia scores three to four months after the treatment were 1 for both groups. However, the same was not true for pain; the drop in median pain score at six months based on the Likert pain scale for the EBRT group was from 7.5 to 4, but for SEMS, the drop in pain score was only from 7 to 5.

Conclusion: EBRT and esophageal SEMS are equally effective in relieving dysphagia, but the same is not true for pain. For patients with SEMS, pain remains a constant concern both for patient and physician. Hence, proper patient selection for either modality should be meticulously weighted based on symptomology.

## Introduction

Esophageal cancer (OC) is the 11th most common cancer worldwide and the seventh most common cause of death, according to Global Cancer Observatory (GLOBOCAN) 2022. Squamous cell cancer (SCC) made up the great majority of esophageal malignancies worldwide for most of the 20th century [1,2]. However, over the last three decades, adenocarcinoma of the esophagus, gastroesophageal junction (GEJ), and gastric cardia have seen a substantial increase in frequency. This discovery was initially noted in Western countries and has more recently been observed in some Eastern countries as well [3]. According to recent reports of the International Agency for Research on Cancer (IARC) and GLOBOCAN 2022 in terms of both incidence and mortality (for both sexes combined), esophageal cancer in India is ranked fifth among all cancer sites [4].

The majority of patients diagnosed with carcinoma of the esophagus have a locally advanced or metastatic disease at presentation [5-8]. Patients with such an advanced disease have a poor quality of life, mainly because of symptoms like dysphagia and pain. Ideal treatment for such patients should provide a rapid and sustainable relief of dysphagia and pain with minimal side effects and toxicity. Various treatment modalities for symptom palliation of such patients who are not candidates for curative treatments are available. Endoluminal stenting, surgery, external beam radiation (EBRT), brachytherapy (BT), chemotherapy (CT), chemoradiotherapy, laser, or photodynamic therapy are some of them. Sometimes ablative procedures like direct injection of alcohol or chemotherapeutic agents are used. Among them, palliative radiotherapy to the esophagus and esophageal stenting are commonly used [9]. This study aimed to compare the efficacy and complication rate of palliative esophageal EBRT and esophageal stent.

## Materials and methods

This was a retrospective observational comparative study. This study is based on observations of 130 patients who were diagnosed with locally advanced unresectable or metastatic esophageal cancer and were not fit to tolerate chemoradiation. They were treated at our institute from January 2017 to December 2023 for symptom palliation with either external beam radiotherapy (EBRT) or placement of self-expanding metallic stents (SEMS). The data were collected by reviewing records entered in patient files, following clearance from the Institutional Ethics Committee of Sher-i-Kashmir Institute of Medical Sciences (SKIMS), Srinagar, India (IEC/SKIMS Protocol #137/2026, date: 05-06-2026). Informed consent was obtained from all patients at the time of registration, stating that without disclosing the patient’s identity, the clinical data may be used in the future for research purposes. All relevant data, including patient details, demographics, clinicopathological details of malignancy, treatment received, treatment side effects, and information on long-term outcomes, were collected.

Inclusion criteria

Histologically confirmed esophageal cancer, dysphagia grade 1 or higher, locally advanced or metastatic stage are included.

Exclusion criteria

Patients were excluded if they had received any cancer-related treatment before, including radiotherapy (RT), a combination of RT and SEMS, or any anticancer systemic treatment (chemotherapy, targeted therapy, or immunotherapy), or if they had a history of any previous malignancy.

Treatment and clinical outcomes

Patients in the RT group received EBRT using photons from either cobalt 60 or high-energy (6 MV) linear accelerators. Patients were treated in supine position using two (anterior/posterior) parallel-opposed fields to a dose of 35Gy in 15 fractions. SEMS placement was generally avoided in patients with tumors involving the cervical esophagus because of technical limitations and the potential for patient discomfort. Tumors involving the gastroesophageal (GE) junction were evaluated on an individual basis, taking into account anatomical feasibility and the risk of gastroesophageal reflux before stent placement. We assessed the efficacy of RT or SEMS by measuring pre- and post-treatment dysphagia and pain scores. Through a manual review of the clinical notes, dysphagia grades and pain scores were calculated. Using a well-established five-point dysphagia scoring system, scores were assigned, with 0 denoting no dysphagia and a score of 4 indicating total inability to swallow [10]. A Likert scale of 0-10 was used for the calculation of pain scores [11].

Pain scores for every patient were recorded before the start of RT or SEMS and for up to one year postintervention. For subgroup analysis, only patients with documented at least one preintervention and one postintervention dysphagia and pain scores were included. The most recent preintervention scores were used as the baseline. Patients’ records were reviewed to identify any adverse effects, esophageal fistula formation, perforation, obstruction, esophagitis, and hemorrhage following EBRT or SEMS.

Statistical analysis

We assessed differences in baseline covariates between the EBRT and SEMS groups using Chi-squared and Wilcoxon tests. The Cox proportional hazards model was used to estimate the hazard ratios (HRs) and 95% confidence intervals (CIs). A two-sided p-value of less than 0.05 was considered significant unless otherwise specified. All statistical analyses were performed using SPSS software (version 24.0, IBM Corp., Armonk, New York, United States).

## Results

Out of 130 patients in our study, 100 (77%) patients received palliative EBRT to the esophagus, and in 30 (23%) patients, SEMS was deployed. Baseline patient characteristics in each group are shown in Table 1.

**Table 1:** Baseline Patient Characteristics ECOG = Eastern Cooperative Oncology Group. Age was compared between groups using an independent-samples t-test. Categorical variables were compared using Pearson's Chi-square test. Test statistics are reported as t-values for continuous variables and χ² values for categorical variables.

Characteristic	Category	Radiotherapy (n = 100), n (%)	Stent (n = 30), n (%)	Test Statistic	p value
Age, median (range), years	–	65 (53-73)	63 (55-70)	t = 0.922	0.361
Gender	Male	52 (52.0)	15 (50.0)	χ² = 4.489	0.106
	Female	48 (48.0)	15 (50.0)		
History of tobacco use	Present	77 (77.0)	24 (80.0)	χ² = 0.009	0.923
	Absent	23 (23.0)	6 (20.0)		
Tumor location in esophagus	Lower third	21 (21.0)	6 (20.0)	χ² = 0.271	0.873
	Middle third	60 (60.0)	17 (56.7)		
	Upper third	19 (19.0)	7 (23.3)		
Histology	Squamous cell carcinoma	88 (88.0)	25 (83.3)	χ² = 0.127	0.722
	Adenocarcinoma	12 (12.0)	5 (16.7)		
ECOG performance status	1	31 (31.0)	9 (30.0)	χ² = 0.021	0.990
	2	37 (37.0)	11 (36.7)		
	3	32 (32.0)	10 (33.3)		

**Figure 1:**
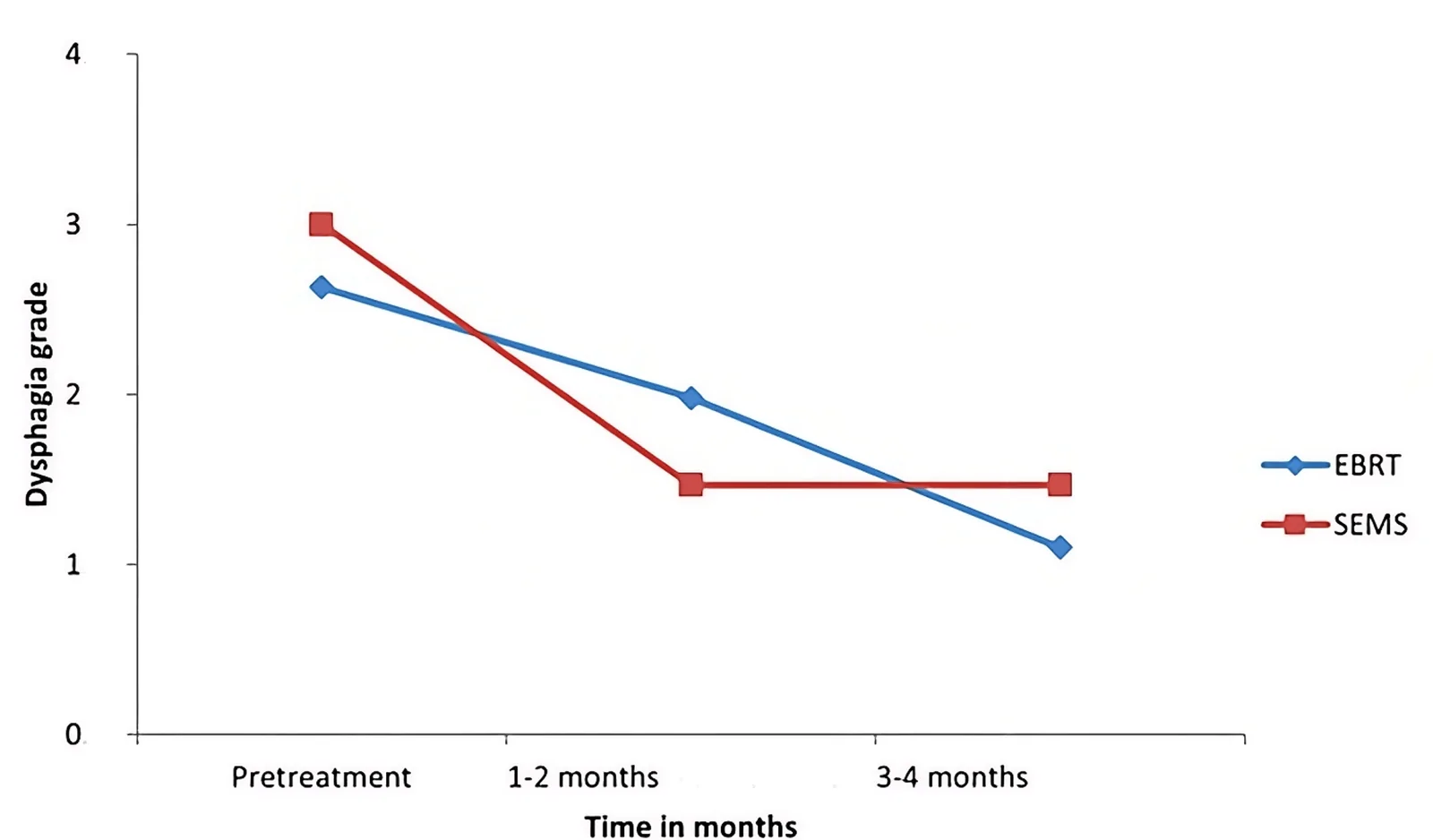
Dysphagia Improvement With EBRT and SEMS EBRT = external beam radiotherapy, SEMS = self-expanding metal stents.

The improvement in dysphagia grade from baseline to one to two months was significantly greater in the EBRT and SEMS groups (Mann-Whitney U test, p < 0.001). However, by three to four months, the degree of dysphagia improvement was comparable between the two groups (p = 0.857). Both treatment modalities were effective in relieving dysphagia. However, SEMS provided immediate relief in almost all patients following complete stent expansion, and this benefit remained stable for approximately four months. In contrast, dysphagia palliation with EBRT was more gradual. Because the total treatment time for completion of EBRT was two weeks, RT also takes time to show its maximum therapeutic effect. We observed that there was continuous improvement in dysphagia even after completion of EBRT (Figure 1).

**Figure 2:**
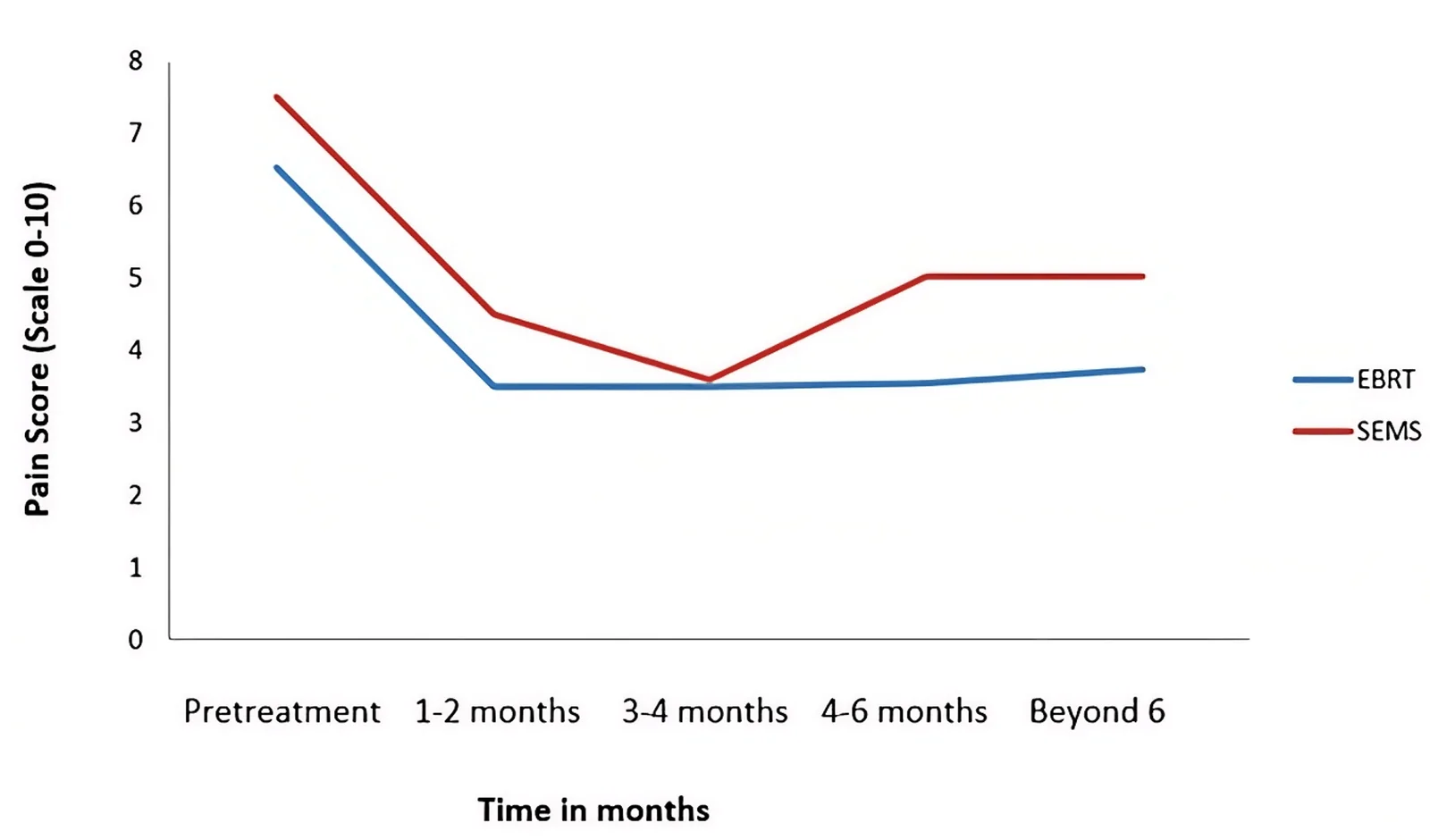
Improvement of Pain With EBRT and SEMS EBRT = external beam radiotherapy, SEMS = self-expanding metal stents.

Patient-reported pain scores demonstrated a significant reduction within one to two months after EBRT, with sustained pain relief throughout the follow-up period. However, analgesic consumption was not systematically documented and therefore could not be used as an objective measure of pain control. Although SEMS insertion caused comparable improvement in pain scores, these patients frequently complained of some degree of retrosternal pain, and some even reported an increase in pain following an initial alleviation (Figure 2).

Stent-induced pain can be due to mechanical distension or pressure necrosis. The majority of patients in both groups tolerated the treatment well, with only a small proportion developing adverse events requiring clinical attention. Such events occurred in 24 patients (24%) in the EBRT group and 10 patients (33.3%) in the SEMS group. The frequencies of the various treatment-related adverse events are summarized in Table 2.

**Table 2:** Side Effects of EBRT and SEMS EBRT = external beam radiotherapy, SEMS = self-expanding metal stents. Overall comparison of side-effect distribution between EBRT and SEMS (χ² = 5.485, p = 0.360), n (%): n is number of patients, % is percent. Fisher's exact test was used because of the low frequency of several adverse events.

Side Effect	EBRT (n = 100), n (%)	SEMS (n = 30), n (%)	p value
Esophagitis	10 (10.0)	2 (6.7)	0.732
Fistula	2 (2.0)	1 (3.3)	0.548
Hemorrhage	4 (4.0)	3 (10.0)	0.200
Obstruction	7 (7.0)	2 (6.7)	1.000
Perforation	1 (1.0)	2 (6.7)	0.133
No adverse effect	76 (76.0)	20 (66.7)	0.346
Overall comparison			0.360

**Figure 3:**
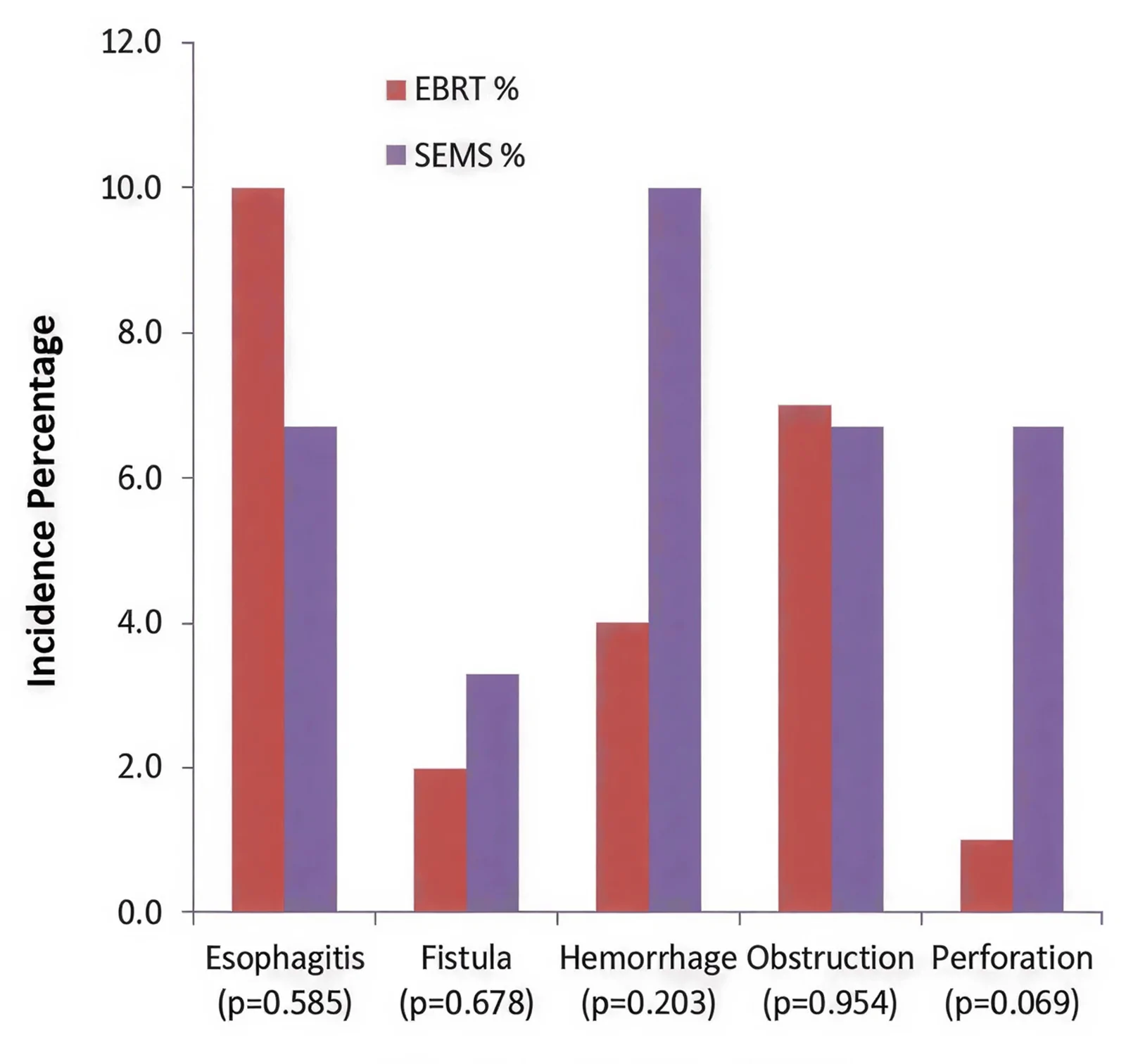
Side Effects of EBRT and SEMS EBRT = external beam radiotherapy, SEMS = self-expanding metal stents.

The side effect profiles of the two modalities were comparable, although their frequency varied considerably (Figure 3).

As depicted in the Figure 3, the common side effects observed in both treatment groups were esophagitis, obstruction, hemorrhage, fistula formation, and perforation. However, in patients who were treated with EBRT, esophagitis was seen as the most common side effect 10 (10%), followed by obstruction 7 (7%), and perforation was the least common 1 (1%) side effect. In patients where SEMS was deployed, hemorrhage was the most common side effect observed 3 (10%), followed by equal frequency of esophagitis, obstruction, and perforation in 2 (6.7%) patients. In EBRT group, perforation was seen in only 1 (1%) patient which is very low when compared with 2 (6.7%) patients in the SEMS group.

## Discussion

Malignant dysphagia is present in about 70% of patients with advanced esophageal cancer, and it adversely affects the quality of life [12,13]. Another frequent and equally distressing symptom in such patients is pain, which is equally linked to a lower quality of life [14]. Pain can be either due to local tumor effects or associated odynophagia. In this context, the most important focus of the treatment in advanced esophageal cancer is relieving symptoms, with EBRT and endoluminal stenting being the most popular treatment modalities. The possible pros and cons of these two therapies can be defined by evaluating their comparative effectiveness in patients with advanced esophageal cancer who have local symptoms. In the present study, we compared the efficacy of EBRT and SEMS for relieving cancer-associated dysphagia and pain.

The most important finding of our study was effective symptom palliation with either of the treatment modalities. Based on the discretion of the treating physician, we observed that the group of patients who received SEMS had more severe preintervention dysphagia than the patients who were treated with either. This observation is in accordance with the published literature [15,16]. It has been demonstrated that stent placement can improve dysphagia scores and restore luminal patency within a day, as opposed to radiotherapy, which takes a longer course to manifest its full effect. Bown observed that the time delay for maximum dysphagia relief with the use of EBRT can be up to six weeks [17]. So, stent placement is often a preferred option when there is severe dysphagia, and the life expectancy of patients is also low [18]. The high rate of dysphagia recurrence because of stent migration and tumor ingrowth significantly restricts the use of the stent. However, radiotherapy has shown durable dysphagia relief in up to 90% of patients [19,20]. We observed that with SEMS, the maximum therapeutic effect was seen for up to one to two months post-procedure, and it remained constant for the next few months of follow-up. However, with EBRT, a different scenario was seen; there was continuous improvement in dysphagia even beyond the initial one to two months postprocedure. A probable explanation for this can be due to the antitumor effect of radiotherapy. Regarding pain, we observed that EBRT caused rapid relief of pain, and the maximum effect was seen one to two months after EBRT, and pain relief was sustained. A meta-analysis done by Xu et al. showed that EBRT significantly reduced tumor-related pain and odynophagia within three to four weeks, with durable symptom control for two to three months or longer in responders [21]. SEMS took a slightly longer time than EBRT to achieve maximum pain control, and this pain control was not sustained for a long time, which aligns with our observation [21].

There was a recurrence of pain, and some amount of pain always remained in SEMS users; they needed additional pain medications on and off. Homs et al. observed that post-stent chest pain or retrosternal pain occurs in 30%-50% of patients. This pain is usually transient but can be severe, requiring opioids [22]. Stent-induced pain tends to peak in the first week after placement and is due to mechanical distension or pressure necrosis. With SEMS, there is no antitumor activity, and continuous tumor growth causes pressure and hence pain. In some patients, after some time, we also observed tumor growth within the lumen of SEMS, where restenting with a second SEMS was done, or other palliative modalities like balloon dilations were used. A similar observation has been seen by Kujawski et al. [23]. Most of the patients in our series tolerated both treatment modalities very well. However, a few patients developed some adverse effects over a varied time span after initiation of treatment. The adverse effects observed in our study were esophagitis, esophageal fistula formation, hemorrhage obstruction, and even perforation. Although the profile of adverse effects of the two modalities was very similar, the frequency of occurrence was different. We observed that esophagitis occurred in a higher number of patients in the EBRT group than in the SEMS group, while other side effects like fistula formation, hemorrhage, and perforation were more common in the SEMS group. However, the incidence of esophageal obstruction was similar in both groups. Our findings are consistent with those of Martin et al., who discovered that patients undergoing stent placement experienced a greater frequency of hemorrhage and perforation as well as a tendency toward an increasing incidence of fistula formation [24].

Limitations

Although we analyze the correlation between treatment modalities, EBRT and SEMS, as our study endpoints, we cannot establish causality. Factors unrelated to esophageal stenting or radiotherapy may contribute to pain, dysphagia, or adverse effects, potentially influencing outcomes independently. Also, analgesic consumption was not systematically documented in this retrospective study; therefore, pain assessment relied solely on patient-reported pain scores. The inclusion of analgesic usage as an objective measure of pain control would have provided a more comprehensive evaluation of treatment efficacy. Despite these limitations, our study offers practical evidence to help both practitioners and patients navigate the choice between palliative RT and esophageal stenting.

## Conclusions

Our results conclude that palliative radiation therapy provides equivalent relief of moderate to severe dysphagia when compared with esophageal stenting in patients with advanced esophageal cancer who are not fit for any curative treatment. The same degree of dysphagia relief, although not so rapid, was observed with EBRT but with a lower risk of toxicity. We also observed that pain control was better and more sustained in such patients receiving EBRT. Prospective trials are necessary to fully understand the relative effectiveness of stent implantation and palliative radiation therapy in advanced esophageal cancer.
